# Vitamin D intake and all-cause and cause-specific mortality in Japanese men and women: the Japan Public Health Center-based prospective study

**DOI:** 10.1007/s10654-023-00968-8

**Published:** 2023-01-31

**Authors:** Akiko Nanri, Tetsuya Mizoue, Atsushi Goto, Mitsuhiko Noda, Norie Sawada, Shoichiro Tsugane, For the Japan Public Health Center-based Prospective Study Group

**Affiliations:** 1 Department of Food and Health Sciences, International College of Arts and Sciences, Fukuoka Women’s University, Fukuoka, Japan; 2 Department of Epidemiology and Prevention, Center for Clinical Sciences, National Center for Global Health and Medicine, Tokyo, Japan; 3 Department of Health Data Science, Graduate School of Data Science, Yokohama City University, Yokohama, Japan; 4 Department of Diabetes, Metabolism and Endocrinology, Ichikawa Hospital, International University of Health and Welfare, Ichikawa, Japan; 5 Division of Cohort Research, National Cancer Center Institute for Cancer Control, Tokyo, Japan; 6 National Institute of Health and Nutrition, National Institutes of Biomedical Innovation, Health and Nutrition, Tokyo, Japan

**Keywords:** Cardiovascular disease, Cohort studies, Japan, Mortality, Pneumonia, Vitamin D

## Abstract

While higher circulating 25-hydroxyvitamin D concentrations have been reported to be associated with decreased risk of all-cause mortality, evidence on dietary vitamin D intake is limited and inconsistent. We investigated whether vitamin D intake is associated with all-cause and cause-specific mortality among Japanese adults. Participants were 42,992 men and 50,693 women who responded to the second survey of the Japan Public Health Center-based Prospective Study (1995–1998) and who were followed up for mortality through 2018. Dietary intake was ascertained using a validated food frequency questionnaire. Hazard ratios of deaths from the second survey to December 2018 were estimated using Cox proportional hazard regression analysis. During follow-up, we identified 22,630 deaths. Overall, the third and fourth quintiles, but not the highest quintile, of vitamin D intake were each associated with a significantly lower risk of all-cause mortality. In subgroups characterized by low sunlight exposure, risk of all-cause mortality decreased linearly with increasing vitamin D intake. The multivariable-adjusted hazard ratios (95% confidence intervals) of all-cause mortality for the highest versus lowest quintile of vitamin D intake were 0.87 (0.79–0.95) in women and 0.88 (0.79–0.97) in residents of higher latitude areas. Lower risk was also observed for all-cause mortality in participants with hypertension and for heart disease mortality in those with higher calcium intake. Higher vitamin D intake was associated with decreased risk of ischemic stroke and pneumonia mortality. Higher dietary vitamin D was associated with a lower risk of mortality among individuals with low sunlight exposure or hypertension. Individuals with potentially low vitamin D may benefit from increasing dietary vitamin D intake for the prevention of premature death.

**Supplementary Information:**

The online version contains supplementary material available at 10.1007/s10654-023-00968-8.

## Introduction

Beside the role of vitamin D in musculoskeletal health, accumulating evidence from experimental and epidemiological studies indicates its protective role against a range of diseases, including cancer, cardiovascular diseases (CVD), autoimmune disease, infectious diseases, and diabetes [1]. A meta-analysis of 73 cohort studies showed that higher circulating concentrations of 25-hydroxivitamin D were associated with a lower risk of mortality from all-cause, CVD, cancer, and some other diseases [2]. More recently, vitamin D deficiency has shown to be associated with an increased risk of death from coronavirus disease 2019 [3]. Evidence from genetic studies supports that the vitamin D-mortality association is causal [4].

In contrast to consistent evidence from studies that measured vitamin D in blood, research on dietary intake of vitamin D did not find any association with all-cause mortality [5–9]. The lack of an association in dietary studies may be ascribed to much greater contribution of cutaneous vitamin D synthesis on sunlight exposure to systemic vitamin D exposure [10], which could conceal the association with dietary intake, if any. Therefore, the effect of dietary vitamin D intake on mortality may be evident only among individuals with lower sunlight exposure (e.g., women, residents of higher latitude regions, people who spend most of their time indoors).

The synergistic effect of vitamin D and calcium has been implicated in the prevention of both skeletal and non-skeletal diseases [11–14]. Accordingly, the impact of vitamin D intake on mortality risk may be pronounced among participants with higher intake of calcium. To date, epidemiological evidence is scarce on the interacting effect of these nutrients on all-cause mortality [8]. Additionally, the benefit of dietary vitamin D may be present only or more clearly observed among high-risk individuals who, for example, are obese, smoke, and have hypertension compared to those who do not. In fact, the Honolulu Heart Program reported an increased risk of death associated with low vitamin D intake among participants with hypertension, but not among those without hypertension [7]. It would thus be of interest to further examine the dietary vitamin D-mortality association in these high-risk individuals.

To address these issues, we prospectively investigated the association of vitamin D intake with the risk of all-cause, cancer, CVD, and respiratory disease mortality in a large-scale population-based cohort study in Japan, where people traditionally consume a large amount of fish, a rich source of vitamin D. We also tested whether the association was modified by the surrogates of sunlight exposure (sex, area of residence, occupation, physical activity), calcium intake, and known risk factors of chronic disease (obesity, smoking, hypertension, and diabetes).

## Methods

### Study design and participants

The Japan Public Health Center-based Prospective (JPHC) Study is a population-based cohort study among 140,420 residents aged 40–69 years in 11 public health center areas across Japan. The detail of the study procedure has been described elsewhere [15]. In short, the first self-administered questionnaire survey was conducted in 1990–1993, where information on height, body weight, medical history, smoking status, physical activity, and other lifestyle factors was obtained. Participants who responded to the questionnaire were regarded as consenting to participate in the study. The second survey was conducted 5 years later (1995–1998) to update information on lifestyle habits and health conditions. The present study set the second survey as baseline because the questionnaire used in the second included comprehensive information on food intakes. This study including the procedure of informed consent was approved by the Institutional Review Board of the National Cancer Center of Japan.

Of the study participants in the first survey (n = 140,420), 102,427 responded to the second survey, including the diet-related portion. We excluded 7766 participants who reported history of cancer, cerebrovascular disease, myocardial infarction, chronic liver disease, and renal disease, at the first or second survey. We further excluded 976 participants who reported extreme total energy intake (outside of the mean ± 3 standard deviations according to sex), leaving 93,685 participants (42,992 men and 50,693 women) for analysis (Supplementary Fig. 1).

### Dietary assessment

A food frequency questionnaire (FFQ) was used to assess the average intake of 147 food and beverage items over the previous year [16]. For most food items, 9 response options were available to describe consumption frequency, which ranged from rarely (< 1 time/month) to ≥ 7 times/day. A standard portion size was specified for each food, and respondents were asked to denote their usual portion size from 3 options (< 0.5 times, standard, or > 1.5 times), for which we assigned 0.5, 1.0, and 1.5, respectively. We calculated the average daily intake of nutrients by multiplying the consumption frequency of each food by its nutrient content per serving and summing the nutrient intake for all food items. The validity of the FFQ was assessed in a subsample using either 14- or 28-day dietary records. Spearman’s correlation coefficients of energy-adjusted intake of vitamin D between FFQ and dietary records were 0.26 to 0.38 for men and women [17].

### Follow-up and outcome

Participants were followed up for residency and vital status through the residential registry. Causes of death were confirmed via death certificates and were defined according to the tenth version of the International Classification of Disease as follows: cancer (C00–C97), CVD (I00–I99), respiratory disease (J10–J18 and J40–J47), pneumonia (J10–J18), and injuries and accidents (V01–Y09 and Y85–Y86). Moreover, CVD mortality was subdivided into heart disease (I20–I52) and stroke (I60–I69), and stroke was subdivided into ischemic stroke (I63), intraparenchymal hemorrhage (I61), and subarachnoid hemorrhage (I60). Cancer was divided by site into lung cancer (C34), colorectal cancer (C18–C20), esophageal cancer (C15), stomach cancer (C16), pancreas cancer (C25), liver cancer (C22), biliary tract cancer (C23–C24), breast cancer (C50), and prostate cancer (C61). Pearson-year of follow-up were calculated for each person from the date of response to the second survey questionnaire to the date of death, emigration from Japan, or the end of follow-up (December 31, 2018 for 9 areas, December 31, 2009 for 1 area, and December 31, 2012 for 1 area), whichever came first.

### Statistical analysis

Participants were divided into quintiles of energy-adjusted vitamin D intake (by residual methods). Baseline characteristics were expressed as mean (standard deviation) and percentage. Cox proportional hazard regression analysis was performed to estimate hazard ratios and 95% CIs of mortality for quintile of vitamin D intake, with the lowest category as the reference. The first model was adjusted for age (years), sex, and study site (11 areas), and the second model was further adjusted for BMI (< 21, 21–22.9, 23–24.9, 25–26.9, or ≥ 27 kg/m^2^), smoking status (never, past, or current with a consumption of < 20 or ≥ 20 cigarettes/day), alcohol consumption (nondrinker, occasional drinker, or drinker with a consumption of < 150, 150–299, 300–449, or ≥ 450 g ethanol/week), history of diabetes mellitus (yes or no), history of hypertension (yes or no), total physical activity [quartile of metabolic equivalent of task (MET) hours/day], occupation (agriculture, forestry, or fishery; salaried, self-employed, or professional; or housework, unemployed, or retired), leisure-time physical activity (< 1 time/month, 1–2 times/month, or ≥ 1 time/week), total energy intake (kcal/day), supplement use (yes or no), green tea consumption (almost never, < 1, 1, 2–3, or ≥ 4 cups/day), coffee consumption (almost never, < 1, 1, or ≥ 2 cups/day), energy-adjusted calcium intake (mg/day), and n − 3 polyunsaturated fatty acid intake (%energy). An indicator variable for missing data was created for each covariate. Trend associations were assessed by treating the median of each quintile of vitamin D intake as a continuous variable. We repeated the analysis after excluding participants who died in the first 5 years of follow-up to minimize the effect of potential occult disease at baseline.

We also analyzed the association between vitamin D intake and mortality by sex, age (< 60 years old or ≥ 60 years old), area of residence (higher latitude area or lower latitude area), calcium intake (< median or ≥ median), BMI (< 25 kg/m^2^ or ≥ 25 kg/m^2^), smoking status in men only (nonsmoker or current smoker), history of hypertension (yes or no), history of diabetes (yes or no), occupation (primary industry or non-primary industry), leisure-time physical activity (< 1 time/week or ≥ 1 time/week), and total physical activity (METs-hours/day, < median or ≥ median). An interaction term, created by multiplying vitamin D intake (quintile) and the above stratifying variables (dichotomous), was added to the model to assess statistical interactions. Two-sided *P* values < 0.05 were regarded as statistically significant. All analyses were performed using Statistical Analysis System (SAS) software version 9.4 (SAS Institute, Cary, NC, USA).

## Results

Baseline characteristics of participants according to quintile of vitamin D intake are shown in Table 1. Participants with higher intake of vitamin D were older, more likely to be women, residents of higher latitude areas, and to report histories of diabetes, and less likely to be smokers and alcohol drinkers compared to those with lower intake. They also had higher calcium, n − 3 polyunsaturated fatty acid, and green tea intake but lower total energy intake and coffee consumption.

**Table 1:** Baseline characteristics according to quintile (Q) of vitamin D intake

	Quintile of vitamin D intake				
	Q1 (low)	Q2	Q3	Q4	Q5 (high)
No. of participants	18,737	18,737	18,737	18,737	18,737
Age, mean ± SD, year	56.7 ± 8.3	55.9 ± 8.0	56.1 ± 7.7	56.8 ± 7.7	58.0 ± 7.6
Sex (women), %	41.0	47.2	54.4	61.1	66.8
Area (higher latitude), %	20.9	32.1	38.0	41.8	44.9
Body mass index, mean ± SD, kg/m^2^	23.8 ± 3.1	23.5 ± 3.1	23.4 ± 3.0	23.4 ± 3.0	23.4 ± 3.1
Current smoker, %	30.3	28.6	25.3	22.4	19.5
Current drinker (≥ 1 time/week), %	45.2	43.0	39.7	35.2	28.9
Total physical activity, mean ± SD, MET-hour/week	33.6 ± 6.4	33.2 ± 6.3	33.1 ± 6.1	33.0 ± 6.1	32.8 ± 6.0
Leisure-time physical activity (≥ 1 time/week), %	20.9	22.9	22.4	22.8	20.9
Occupation (primary industries), %	22.5	17.6	16.5	16.9	18.9
Total energy intake, mean ± SD, kcal	2066 ± 839	2064 ± 720	2051 ± 698	2020 ± 680	1930 ± 671
History of hypertension, %	18.5	17.1	17.9	18.9	21.0
History of diabetes, %	4.5	4.6	4.8	5.4	6.0
Supplement use, %	11.0	13.3	14.2	14.1	12.8
Vitamin D intake^a^, mean ± SD, µg/day	3.6 ± 1.3	6.6 ± 0.7	9.2 ± 0.8	12.5 ± 1.2	21.0 ± 7.7
Calcium intake^a^, mean ± SD, mg/day	427 ± 226	500 ± 240	524 ± 233	537 ± 214	546 ± 204
n-3 PUFA intake, mean ± SD, %energy	0.76 ± 0.29	0.93 ± 0.25	1.08 ± 0.25	1.25 ± 0.27	1.62 ± 0.47
Green tea consumption (≥ 1 cup/day), %	48.2	56.1	62.3	65.0	65.8
Coffee consumption (≥ 1 cup/day), %	39.0	39.8	37.4	34.8	28.5

*PUFA* polyunsaturated fatty acid, *MET* metabolic equivalent of task, *SD* standard deviation

^a^Adjusted for total energy intake by using residual method

During 1,768,746 person-years of follow-up (mean 18.9 years), we identified 22,630 all-cause deaths, 8138 cancer deaths, 6031 CVD deaths (including 3185 heart disease deaths and 2300 stroke deaths), and 1871 respiratory disease deaths (including 1493 pneumonia deaths).

Hazard ratios of mortality according to quintile of vitamin D intake are shown in Table 2. There was a curvilinear association between vitamin D intake and all-cause mortality, showing a significantly lower risk for the third and fourth quintile of vitamin D. As regards major causes of death, risk for CVD, heart disease, and stroke mortality tended to decrease with increasing vitamin D intake, although the trend was not significant. The second through fourth quintiles of vitamin D intake were each significantly associated with decreased risk of stroke mortality. Regarding stroke subtype, vitamin D intake was associated with a decreased risk of ischemic stroke mortality (*P* for trend = 0.029); the multivariable-adjusted hazard ratio for the highest versus lowest quintile of vitamin D intake was 0.62 (95% CI 0.44–0.88) (Supplementary Table 1). The hazard ratios of pneumonia mortality for the third, fourth, and highest quintile of vitamin D intake were 18–21% significantly lower than those of the lowest quintile after adjustment for covariates (*P* for trend = 0.09) (Table 2); the trend association was strengthened after excluding participants who died during the first 5 years of follow-up (*P* for trend = 0.033). For all-cause and cause-specific mortality except pneumonia mortality, the results did not materially change after excluding those who died during the first 5 years of follow-up (data not shown). Risk of death from total cancer or from specific cancer was not associated with vitamin D intake (Table 2 and Supplementary Table 1).

**Table 2:** Hazard ratios (95% CI) for mortality according to quintile (Q) of vitamin D intake

	Quintile of vitamin D intake					*P* for trend^a^
	Q1 (low)	Q2	Q3	Q4	Q5 (high)	
Person-year of follow-up	350,457	351,123	353,408	356,467	357,291	
All-cause						
No. of deaths	5160	4353	4134	4221	4762	
Adjusted HR^b^ (95% CI)	1.00 (ref)	0.92 (0.89–0.96)	0.89 (0.85–0.93)	0.88 (0.84–0.92)	0.93 (0.89–0.97)	0.021
Adjusted HR^c^ (95% CI)	1.00 (ref)	0.96 (0.92–1.002)	0.94 (0.90–0.99)	0.93 (0.89–0.98)	0.96 (0.90–1.02)	0.29
Cancer						
No. of deaths	1787	1650	1556	1499	1646	
Adjusted HR^b^ (95% CI)	1.00 (ref)	0.99 (0.93–1.06)	0.96 (0.89–1.03)	0.91 (0.85–0.98)	0.98 (0.91–1.05)	0.32
Adjusted HR^c^ (95% CI)	1.00 (ref)	1.02 (0.95–1.10)	1.00 (0.93–1.09)	0.96 (0.89–1.05)	1.03 (0.93–1.14)	0.78
Cardiovascular disease						
No. of deaths	1382	1118	1071	1137	1323	
Adjusted HR^b^ (95% CI)	1.00 (ref)	0.88 (0.81–0.95)	0.84 (0.77–0.91)	0.84 (0.77–0.92)	0.89 (0.82–0.97)	0.088
Adjusted HR^c^ (95% CI)	1.00 (ref)	0.92 (0.85–1.003)	0.90 (0.82–0.99)	0.91 (0.82–0.997)	0.91 (0.81–1.02)	0.26
Heart disease						
No. of deaths	745	591	568	613	668	
Adjusted HR^b^ (95% CI)	1.00 (ref)	0.89 (0.79–0.99)	0.86 (0.77–0.97)	0.89 (0.79–0.996)	0.88 (0.78–0.99)	0.13
Adjusted HR^c^ (95% CI)	1.00 (ref)	0.93 (0.83–1.04)	0.92 (0.81–1.04)	0.94 (0.82–1.08)	0.87 (0.74–1.02)	0.16
Stroke						
No. of deaths	523	419	413	412	533	
Adjusted HR^b^ (95% CI)	1.00 (ref)	0.81 (0.71–0.93)	0.78 (0.68–0.89)	0.72 (0.63–0.83)	0.85 (0.74–0.97)	0.11
Adjusted HR^c^ (95% CI)	1.00 (ref)	0.87 (0.76–0.998)	0.85 (0.74–0.99)	0.80 (0.68–0.94)	0.90 (0.75–1.08)	0.54
Respiratory disease						
No. of deaths	460	345	336	332	398	
Adjusted HR^b^ (95% CI)	1.00 (ref)	0.86 (0.74–0.99)	0.85 (0.73–0.98)	0.81 (0.69–0.94)	0.88 (0.76–1.03)	0.26
Adjusted HR^c^ (95% CI)	1.00 (ref)	0.88 (0.76–1.02)	0.87 (0.74–1.03)	0.82 (0.69–0.98)	0.84 (0.68–1.03)	0.16
Pneumonia						
No. of deaths	352	272	264	276	329	
Adjusted HR^b^ (95% CI)	1.00 (ref)	0.83 (0.70–0.97)	0.78 (0.66–0.93)	0.77 (0.65–0.91)	0.83 (0.70–0.98)	0.11
Adjusted HR^c^ (95% CI)	1.00 (ref)	0.86 (0.73–1.02)	0.82 (0.68–0.98)	0.79 (0.65–0.96)	0.79 (0.62–0.99)	0.09
Injury						
No. of deaths	313	274	255	304	293	
Adjusted HR^b^ (95% CI)	1.00 (ref)	0.90 (0.76–1.06)	0.85 (0.72–1.01)	1.02 (0.86–1.20)	0.97 (0.81–1.15)	0.69
Adjusted HR^c^ (95% CI)	1.00 (ref)	0.99 (0.84–1.18)	0.97 (0.80–1.17)	1.16 (0.95–1.41)	1.07 (0.84–1.36)	0.36
Other causes						
No. of deaths	1218	968	916	949	1103	
Adjusted HR^b^ (95% CI)	1.00 (ref)	0.91 (0.84–0.99)	0.89 (0.81–0.97)	0.89 (0.81–0.97)	0.94 (0.86–1.03)	0.44
Adjusted HR^c^ (95% CI)	1.00 (ref)	0.94 (0.86–1.02)	0.92 (0.84–1.02)	0.92 (0.83–1.02)	0.93 (0.82–1.06)	0.45

*CI* confidence interval, *HR* hazard ratio, *ref* reference

^a^Based on Cox proportional hazards model, assigning median intake to the quintile of vitamin D intake

^b^Adjusted for age (year), sex, and study area (11 areas)

^c^Additionally adjusted for body mass index (< 21, 21–22.9, 23–24.9, 25–26.9, or ≥ 27 kg/m^2^), smoking status (never, past, or current with a consumption of < 20 or ≥ 20 cigarettes/day), alcohol consumption (nondrinker, occasional drinker, or drinker with a consumption of < 150, 150–299, 300–449, or ≥ 450 g ethanol/week), history of diabetes mellitus (yes or no), history of hypertension (yes or no), total physical activity (quartile of metabolic equivalent task hours/day), occupation (agriculture, forestry, or fishery; salaried, self-employed, or professional; or housework, unemployed, or retired), leisure-time physical activity (< 1 time/month, 1–2 times/month, or ≥ 1 time/week), total energy intake (kcal/day), supplement use (yes or no), green tea consumption (almost never, < 1, 1, 2–3, or ≥ 4 cups/day), coffee consumption (almost never, < 1, 1, or ≥ 2 cups/day), energy-adjusted calcium intake (mg/day), and n − 3 polyunsaturated fatty acid intake (%energy)

**Fig. 1:**
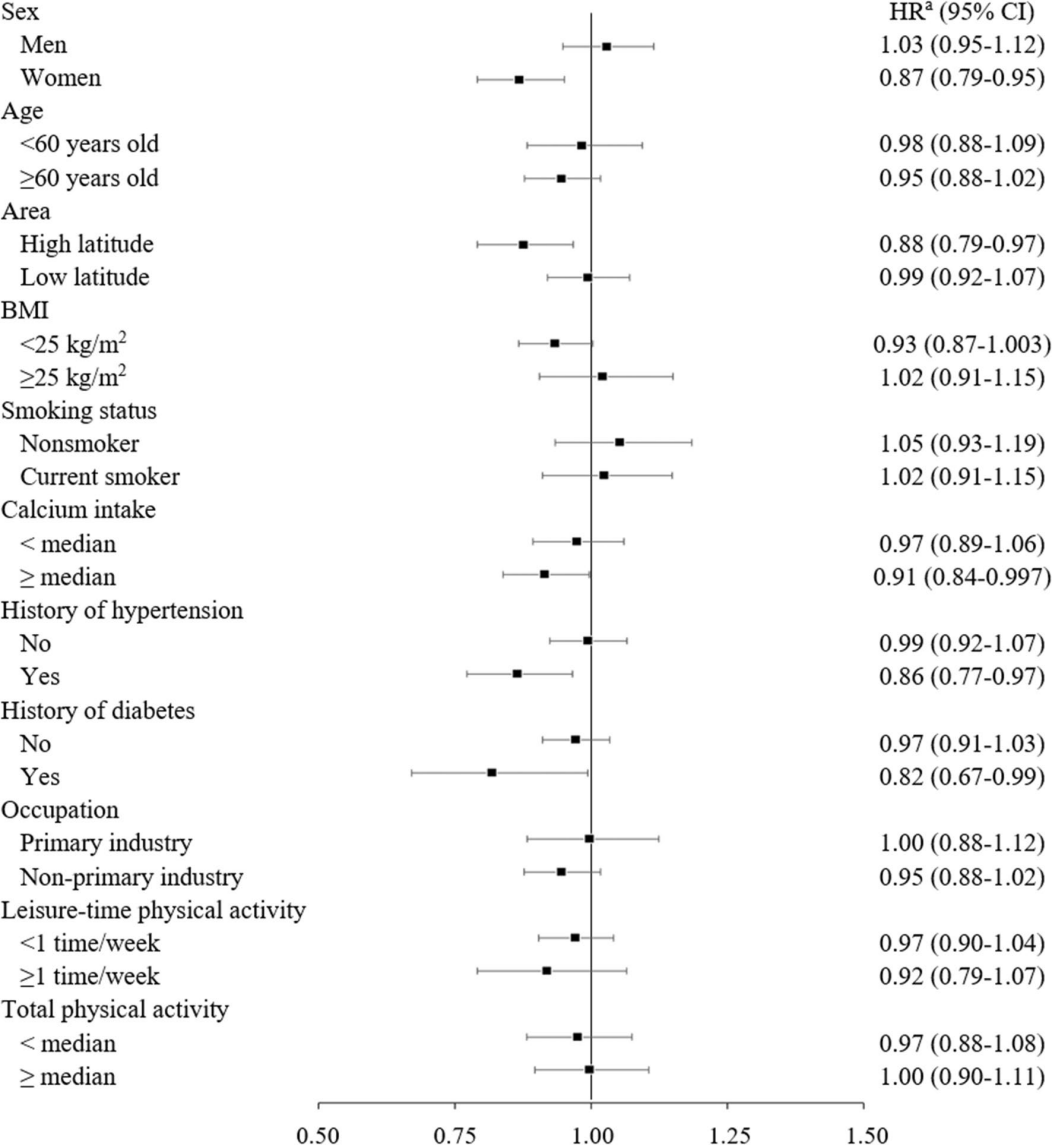
Hazard ratio (95% confidence interval) for all-cause mortality of the highest versus lowest quintile of vitamin D intake in subgroups. *BMI* body mass index, *CI* confidence interval, *HR* hazard ratio. ^a^Adjusted for age, sex, study area, body mass index, smoking status, alcohol consumption, history of diabetes mellitus, history of hypertension, total physical activity, occupation, leisure-time physical activity, total energy intake, supplement use, green tea consumption, coffee consumption, energy-adjusted calcium intake, and n − 3 polyunsaturated fatty acid intake

**Fig. 2:**
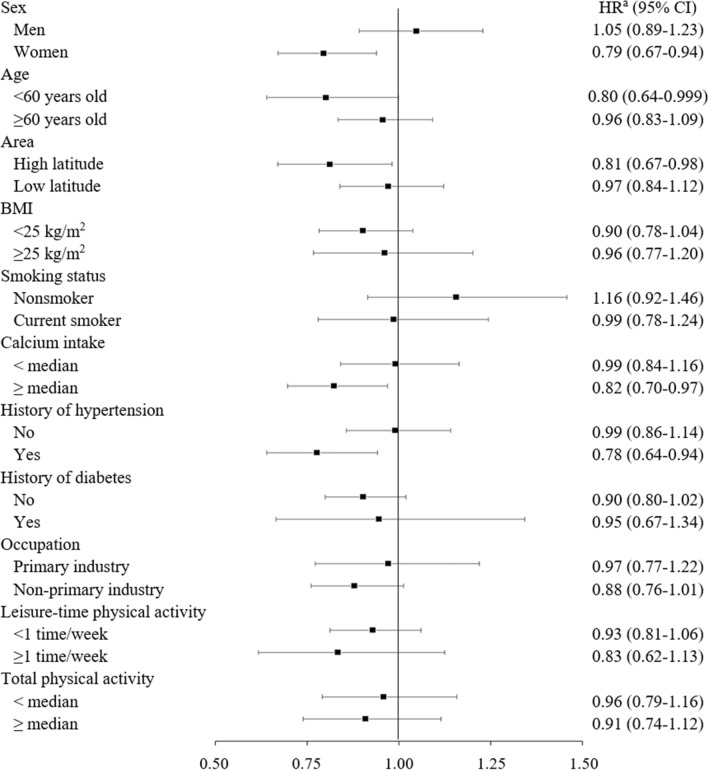
Hazard ratio (95% confidence interval) for cardiovascular disease mortality of the highest versus lowest quintile of vitamin D intake in subgroups. *BMI* body mass index, *CI* confidence interval, *HR* hazard ratio. ^a^Adjusted for age, sex, study area, body mass index, smoking status, alcohol consumption, history of diabetes mellitus, history of hypertension, total physical activity, occupation, leisure-time physical activity, total energy intake, supplement use, green tea consumption, coffee consumption, energy-adjusted calcium intake, and n − 3 polyunsaturated fatty acid intake

**Fig. 3:**
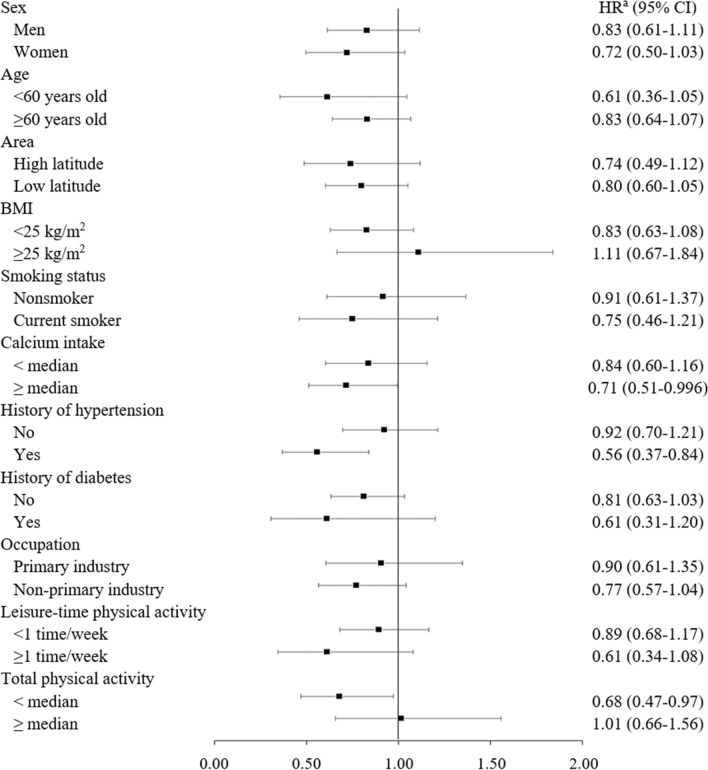
Hazard ratio (95% confidence interval) for pneumonia mortality of the highest versus lowest quintile of vitamin D intake in subgroups. *BMI* body mass index, *CI* confidence interval, *HR* hazard ratio. ^a^Adjusted for age, sex, study area, body mass index, smoking status, alcohol consumption, history of diabetes mellitus, history of hypertension, total physical activity, occupation, leisure-time physical activity, total energy intake, supplement use, green tea consumption, coffee consumption, energy-adjusted calcium intake, and n − 3 polyunsaturated fatty acid intake

In stratified analyses, a lower risk of death from all-cause, CVD, and pneumonia associated with high vitamin D intake was observed in women, residents of higher latitude areas, and those with hypertension, but not in their counterparts (Figs. 1, 2 and 3 and Supplementary Table 2). For example, the multivariable-adjusted hazard ratios (95% CI) of mortality from all-cause, CVD, and pneumonia for the highest versus lowest quintile of vitamin D intake were 0.87 (0.79–0.95), 0.79 (0.67–0.94), and 0.72 (0.50–1.03), respectively in women. In men, the corresponding values were 1.03 (0.95–1.12), 1.05 (0.89–1.23), and 0.83 (0.61–1.11). Among participants with higher intake of calcium, the hazard ratios of all-cause, CVD, pneumonia mortality in the highest quintile of vitamin D were significantly decreased than those in the lowest quintile, with the trend association being statistically significant for CVD mortality. In addition, higher vitamin D intake was associated with a lower risk of heart disease mortality in participants with higher, but not lower, intake of calcium, while it was associated with a lower risk of ischemic stroke mortality in those with lower, but not higher, intake of calcium.

## Discussion

In this large-scale, population-based, prospective study among Japanese, modestly higher intake of vitamin D (third and fourth quintiles) was associated with a decreased risk of all-cause mortality. Moreover, a significantly lower risk of all-cause mortality associated with higher vitamin D intake was observed among women, residents of higher latitude areas, participants with higher intake of calcium, and those with hypertension. Cause-specific analysis showed that vitamin D intake was associated with decreased risk of death from ischemic stroke and pneumonia, but not overall or any type of cancer. To our knowledge, the present study is the first to examine the association of vitamin D intake with all-cause mortality in Asia.

Previous studies reported no significant association between dietary vitamin D intake and all-cause mortality [5–9]. In our hypothesis-oriented analyses stratified by correlates of sunlight exposure, vitamin D intake was associated with decreased risk of all-cause mortality among participants with low sunlight exposure (women and residents of higher latitude areas), but not among those with high sunlight exposure (men and residents of lower latitude areas). This finding supports the hypothesis that high vitamin D levels decreases the risk of death among those with less sunlight exposure. Mizoue et al. [18] reported that higher dietary vitamin D was significantly associated with decreased risk of colorectal cancer only among those who had fewer chances for sunlight exposure at work or in leisure. Studies linking dietary vitamin D to morbidity/mortality should therefore consider sunlight exposure.

Diet-based studies have shown no association between vitamin D intake and CVD events [5, 9, 19, 20]. Since vitamin D enhances intestinal absorption of calcium and renal conservation of the absorbed calcium [10], a concern has been raised about the detrimental effect of high vitamin D and calcium intakes on CVD through vascular calcification. In the present study, however, the risk of death from CVD and heart disease associated with high vitamin D intake was decreased among participants with higher, but not lower, intake of calcium, suggesting that calcium may augment the cardioprotective effect of vitamin D. Studies show that the association between calcium intake and CVD risk is U-shaped [21]. In a population with low calcium intake such as Japanese, dietary vitamin D may prevent CVD among those who consume adequate amount of calcium.

Stroke mortality is higher in north-west region of Japan, where sunlight during winter months is very weak. In the present study, higher vitamin D intake was associated with significantly lower risk of death from ischemic stroke. This association was more evident among residents in higher latitude and participants with hypertension. In contrast, the Japan Collaborative Cohort Study reported decreased risk of mortality from intraparenchymal hemorrhage, but not ischemic stroke [22]. However, our finding is in line with that of the Honolulu Heart Study among Japanese–American, showing significantly lower risk of thromboembolic stroke, but not hemorrhagic stroke, associated with high vitamin D intake [23]. Similarly, higher concentrations of 25-hydroxyvitamin D have been linked to a decreased risk of ischemic stroke but not hemorrhagic stroke in a meta-analysis [24]. Our finding suggests an important role of dietary vitamin D in the protection against ischemic stroke for residents in higher latitude or those with hypertension. Further studies are required to clarify the mechanisms behind the differential association of vitamin D with stroke types.

We found no evidence of a lower risk of cancer death associated with high vitamin D intake among participants overall or by subgroup. This finding agrees with those of a small cohort study in Australia [5]. In contrast to the results of these dietary studies, prospective studies (including that from the JPHC study [25]) that measured circulating 25-hydroxyvitamin D [26] and vitamin D supplementation trials [27, 28] have reported data compatible with the protective role of vitamin D against cancer. The discrepancy between dietary and other study results may be explained by the amount of vitamin D required for cancer prevention. For example, in the Fukuoka Colorectal Cancer Study [18], decreased risk of colorectal cancer associated with vitamin D intake was observed only in those in the higher-half of the highest quintile of vitamin D intake. Extremely high intake of vitamin D, which is impossible to achieve from dietary intake alone, may be required for cancer prevention.

In the present study, the risk of pneumonia mortality was significantly decreased in participants with higher intake of vitamin D. This finding is compatible with that of a meta-analysis of studies that measured circulating vitamin D [29]. In contrast, the Nurses’ Health Study II reported that vitamin D intake from diet and supplement was not associated with the risk of community-acquired pneumonia [30]. Similarly, in a large Dutch case-control study [31], the use of vitamin D supplementation was not associated with the risk of pneumonia. Although the reason for the inconsistent findings is unclear, it might be partly attributable to the difference in BMI across studies. A meta-analysis of vitamin D supplementation and acute respiratory infections demonstrated an effect of vitamin D supplementation among participants with BMI < 25 kg/m^2^, but not among those with BMI ≥ 25 kg/m^2^ [32], suggesting that obesity may be a modifier of the association between vitamin D and pneumonia mortality. Given that Japanese population have a lower BMI than Western population, the association between vitamin D intake and pneumonia mortality would be more clearly observed among the Japanese.

Major strengths of the present study include its large sample size, population-based prospective design, long follow-up period, relatively low loss to follow-up rate, and adjustment for important confounders. However, several limitations of the present study warrant mention. First, the validity of vitamin D intake assessed by the FFQ was not high. In addition, dietary intake was assessed at only one time point. Repeated dietary assessment using a FFQ with higher precision would likely provide a better estimate of exposure status. Second, we excluded participants who did not respond to the second survey. We cannot rule out the possibility of selection bias due to the exclusions. Third, due to the small number of participants in each subgroup, the results of subgroup analyses may be due to chance and should be interpreted with caution. Fourth, we cannot rule out the possibility of unmeasured and residual confounding. Finally, the present findings may not be applicable to non-Japanese populations.

In conclusion, higher vitamin D intake was associated with a significantly lower risk of death from all-cause among individuals characterized by low sunlight exposure. Higher vitamin D intake was associated with a lower risk of death from ischemic stroke and pneumonia. Further studies are required to specify the individuals who can receive health benefit from increasing dietary vitamin D.

## Supplementary Information

Below is the link to the electronic supplementary material.

Supplementary file1 (DOCX 83 KB)

## Abbreviations

CVD: Cardiovascular disease
FFQ: Food frequency questionnaire
JPHC Study: Japan Public Health Center-based prospective study
MET: Metabolic equivalent of task
