# Supplementary material for: Vitamin D intake and all-cause and cause-specific mortality in Japanese men and women: the Japan Public Health Center-based prospective study
Source: Eur J Epidemiol. 2023 Jan 31;38(3):291–300. doi: 10.1007/s10654-023-00968-8 (PMC9887248; doi:10.1007/s10654-023-00968-8)
Supplement: Supplementary file 1 — Supplementary file1 (DOCX 83 KB) [file 10654_2023_968_MOESM1_ESM.docx]

**Vitamin D intake and all-cause and cause-specific mortality in Japanese men and women: The Japan Public Health Center-based Prospective Study**

European Journal of Epidemiology

Nanri A, Mizoue T, Goto A, Noda M, Sawada N, Tsugane S, for the Japan Public Health Center-based Prospective Study Group

Corresponding author: Nanri A; Department of Food and Health Sciences, International College of Arts and Sciences, Fukuoka Women's University, Japan; nanri@fwu.ac.jp

140,420 residents enrolled in JPHC Study health center areas

Non-Japanese nationalities, late report of migration before the start of the follow-up period (1990-1993), incorrect birth date, or duplicate registration (n = 278)

Participants who died, moved, refused follow-up, or lost to follow-up before 1995-1998 (n = 5605)

135,537 participants

Participants who did not complete the second survey questionnaire including the diet-related portion (n = 33,110)

102,427 participants

Participants with history of cancer, cerebrovascular disease, myocardial infarction, chronic liver disease, and renal disease at the first or second survey (n = 7766)

94,661 participants

Participants who reported extreme total energy intake (n = 976)

93,685 participants (42,992 men and 50,693 women)

**Supplementary Fig. 1**  Flowchart of the study participants

**Supplementary Table 1** Hazard ratios (95% confidence intervals) for mortality from site-specific cancer and stroke subtype according to quintile (Q) of vitamin D intake

|  | Quintile of vitamin D intake | | | | |  |
| --- | --- | --- | --- | --- | --- | --- |
|  | Q1 (low) | Q2 | Q3 | Q4 | Q5 (high) | *P* for trend^a^ |
| Site-specific cancer |  |  |  |  |  |  |
| Lung cancer |  |  |  |  |  |  |
| No. of deaths | 401 | 341 | 330 | 300 | 331 |  |
| Adjusted HR^b^ (95% CI) | 1.00 (ref) | 1.00 (0.86-1.17) | 1.08 (0.91-1.27) | 1.04 (0.86-1.25) | 1.18 (0.95-1.48) | 0.13 |
| Colorectal cancer |  |  |  |  |  |  |
| No. of deaths | 199 | 238 | 174 | 178 | 204 |  |
| Adjusted HR^b^ (95% CI) | 1.00 (ref) | 1.36 (1.11-1.66) | 1.03 (0.82-1.30) | 1.03 (0.80-1.32) | 1.08 (0.81-1.45) | 0.66 |
| Esophageal cancer |  |  |  |  |  |  |
| No. of deaths | 79 | 56 | 43 | 46 | 52 |  |
| Adjusted HR^b^ (95% CI) | 1.00 (ref) | 0.82 (0.57-1.19) | 0.70 (0.46-1.07) | 0.80 (0.50-1.28) | 0.99 (0.56-1.74) | 0.81 |
| Stomach cancer |  |  |  |  |  |  |
| No. of deaths | 222 | 215 | 215 | 225 | 222 |  |
| Adjusted HR^b^ (95% CI) | 1.00 (ref) | 0.96 (0.79-1.17) | 0.97 (0.79-1.21) | 1.03 (0.82-1.30) | 1.07 (0.81-1.43) | 0.45 |
| Pancreas cancer |  |  |  |  |  |  |
| No. of deaths | 139 | 135 | 154 | 140 | 155 |  |
| Adjusted HR^b^ (95% CI) | 1.00 (ref) | 0.99 (0.77-1.28) | 1.11 (0.86-1.45) | 0.97 (0.72-1.30) | 1.02 (0.72-1.44) | 0.95 |
| Liver cancer |  |  |  |  |  |  |
| No. of deaths | 138 | 100 | 93 | 93 | 114 |  |
| Adjusted HR^b^ (95% CI) | 1.00 (ref) | 0.78 (0.59-1.02) | 0.75 (0.56-1.01) | 0.74 (0.53-1.02) | 0.84 (0.57-1.24) | 0.65 |
| Biliary tract cancer |  |  |  |  |  |  |
| No. of deaths | 73 | 67 | 61 | 69 | 64 |  |
| Adjusted HR^b^ (95% CI) | 1.00 (ref) | 1.01 (0.71-1.44) | 0.93 (0.63-1.37) | 1.00 (0.66-1.50) | 0.81 (0.49-1.35) | 0.40 |
| Breast cancer |  |  |  |  |  |  |
| No. of deaths | 24 | 33 | 37 | 37 | 46 |  |
| Adjusted HR^b^ (95% CI) | 1.00 (ref) | 1.13 (0.65-1.97) | 1.05 (0.59-1.88) | 0.90 (0.48-1.68) | 0.97 (0.47-1.97) | 0.71 |
| Prostate cancer |  |  |  |  |  |  |
| No. of deaths | 61 | 64 | 49 | 43 | 39 |  |
| Adjusted HR^b^ (95% CI) | 1.00 (ref) | 1.48 (1.01-2.16) | 1.33 (0.86-2.08) | 1.33 (0.80-2.19) | 1.37 (0.74-2.56) | 0.58 |
| Stroke subtype |  |  |  |  |  |  |
| Ischemic stroke |  |  |  |  |  |  |
| No. of deaths | 152 | 111 | 105 | 122 | 134 |  |
| Adjusted HR^b^ (95% CI) | 1.00 (ref) | 0.76 (0.59-0.99) | 0.69 (0.52-0.92) | 0.72 (0.54-0.96) | 0.62 (0.44-0.88) | 0.029 |
| Intraparenchymal hemorrhage |  |  |  |  |  |  |
| No. of deaths | 145 | 113 | 128 | 117 | 153 |  |
| Adjusted HR^b^ (95% CI) | 1.00 (ref) | 0.96 (0.74-1.25) | 1.18 (0.89-1.55) | 1.08 (0.80-1.47) | 1.37 (0.96-1.96) | 0.05 |
| Subarachnoid hemorrhage |  |  |  |  |  |  |
| No. of deaths | 60 | 56 | 60 | 76 | 98 |  |
| Adjusted HR^b^ (95% CI) | 1.00 (ref) | 0.89 (0.61-1.30) | 0.89 (0.60-1.33) | 1.04 (0.69-1.56) | 1.14 (0.71-1.83) | 0.30 |

Abbreviations: CI, confidence interval; HR, hazard ratio; ref, reference.

^a^Based on Cox proportional hazards model, assigning median intake to the quintile of vitamin D intake.

^b^Adjusted for age (year), sex, study area (11 areas), body mass index (<21, 21–22.9, 23–24.9, 25–26.9, or ≥27 kg/m^2^), smoking status (never, past, or current with a consumption of <20 or ≥20 cigarettes/day), alcohol consumption (nondrinker, occasional drinker, or drinker with a consumption of <150, 150–299, 300–449, or ≥450 g ethanol/week), history of diabetes mellitus (yes or no), history of hypertension (yes or no), total physical activity (quartile of metabolic equivalent task hours/day), occupation (agriculture, forestry, or fishery; salaried, self-employed, or professional; or housework, unemployed, or retired), leisure-time physical activity (<1 time/month, 1–2 times/month, or ≥1 time/week), total energy intake (kcal/day), supplement use (yes or no), green tea consumption (almost never, <1, 1, 2–3, or ≥4 cups/day), coffee consumption (almost never, <1, 1, or ≥2 cups/day), energy-adjusted calcium intake (mg/day), and n-3 polyunsaturated fatty acid intake (%energy).

**Supplementary Table 2** Multivariable-adjusted^a^ hazard ratios (95% confidence intervals) for mortality according to quintile (Q) of vitamin D intake by sex, age, area, history of hypertension, and calcium intake

|  | Q1 (low) | Q3 | Q5 (high) | Trend *P*^2^ |  | Q1 (low) | Q3 | Q5 (high) | Trend *P*^b^ | Interaction *P* |
| --- | --- | --- | --- | --- | --- | --- | --- | --- | --- | --- |
|  | Men (n = 42,992) | | | |  | Women (n = 50,693) | | | |  |
| No. of participants | 11051 | 8536 | 6228 |  |  | 7686 | 10201 | 12509 |  |  |
| All-cause mortality | 1.00 (ref) | 0.95 (0.90-1.01) | 1.03 (0.95-1.12) | 0.31 |  | 1.00 (ref) | 0.93 (0.86-1.01) | 0.87 (0.79-0.95) | 0.001 | 0.053 |
| Cancer | 1.00 (ref) | 0.97 (0.88-1.06) | 1.07 (0.93-1.22) | 0.61 |  | 1.00 (ref) | 1.05 (0.92-1.21) | 0.98 (0.83-1.15) | 0.57 | 0.84 |
| Cardiovascular disease | 1.00 (ref) | 0.93 (0.83-1.05) | 1.05 (0.89-1.23) | 0.22 |  | 1.00 (ref) | 0.88 (0.76-1.02) | 0.79 (0.67-0.94) | 0.003 | 0.24 |
| Heart disease | 1.00 (ref) | 1.01 (0.85-1.19) | 1.15 (0.92-1.44) | 0.11 |  | 1.00 (ref) | 0.84 (0.69-1.01) | 0.65 (0.52-0.82) | <0.001 | 0.052 |
| Stroke | 1.00 (ref) | 0.84 (0.70-1.02) | 0.89 (0.69-1.16) | 0.80 |  | 1.00 (ref) | 0.92 (0.72-1.17) | 0.95 (0.72-1.24) | 0.41 | 0.58 |
| Ischemic stroke | 1.00 (ref) | 0.71 (0.50-1.03) | 0.56 (0.34-0.92) | 0.11 |  | 1.00 (ref) | 0.68 (0.43-1.06) | 0.69 (0.42-1.14) | 0.13 | 0.45 |
| Intraparenchymal hemorrhage | 1.00 (ref) | 1.15 (0.82-1.62) | 1.42 (0.89-2.26) | 0.060 |  | 1.00 (ref) | 1.27 (0.80-2.04) | 1.39 (0.79-2.43) | 0.60 | 0.41 |
| Respiratory disease | 1.00 (ref) | 0.91 (0.75-1.11) | 0.88 (0.68-1.15) | 0.50 |  | 1.00 (ref) | 0.80 (0.60-1.06) | 0.74 (0.53-1.04) | 0.12 | 0.12 |
| Pneumonia | 1.00 (ref) | 0.86 (0.69-1.08) | 0.83 (0.61-1.11) | 0.32 |  | 1.00 (ref) | 0.74 (0.54-1.01) | 0.72 (0.50-1.03) | 0.13 | 0.22 |
|  |  |  |  |  |  |  |  |  |  |  |
|  | <60 years old (n = 59,412) | | | |  | ≥60 years old (n = 34,273) | | | |  |
| No. of participants | 11713 | 12472 | 10707 |  |  | 7024 | 6265 | 8030 |  |  |
| All-cause mortality | 1.00 (ref) | 0.98 (0.91-1.06) | 0.98 (0.88-1.09) | 0.71 |  | 1.00 (ref) | 0.93 (0.87-0.98) | 0.95 (0.88-1.02) | 0.32 | 0.055 |
| Cancer | 1.00 (ref) | 1.09 (0.96-1.22) | 1.14 (0.97-1.34) | 0.20 |  | 1.00 (ref) | 0.94 (0.85-1.04) | 0.95 (0.84-1.09) | 0.41 | 0.080 |
| Cardiovascular disease | 1.00 (ref) | 0.97 (0.83-1.14) | 0.80 (0.64-0.999) | 0.045 |  | 1.00 (ref) | 0.87 (0.78-0.98) | 0.96 (0.83-1.09) | 0.97 | 0.014 |
| Heart disease | 1.00 (ref) | 0.95 (0.76-1.18) | 0.77 (0.56-1.05) | 0.10 |  | 1.00 (ref) | 0.92 (0.79-1.07) | 0.92 (0.76-1.11) | 0.53 | 0.022 |
| Stroke | 1.00 (ref) | 0.97 (0.76-1.26) | 0.81 (0.57-1.15) | 0.21 |  | 1.00 (ref) | 0.80 (0.67-0.96) | 0.94 (0.75-1.16) | 0.89 | 0.10 |
| Ischemic stroke | 1.00 (ref) | 0.86 (0.47-1.57) | 0.55 (0.24-1.27) | 0.13 |  | 1.00 (ref) | 0.64 (0.47-0.89) | 0.63 (0.43-0.93) | 0.080 | 0.70 |
| Intraparenchymal hemorrhage | 1.00 (ref) | 1.42 (0.94-2.13) | 1.02 (0.56-1.85) | 0.81 |  | 1.00 (ref) | 1.01 (0.69-1.48) | 1.64 (1.05-2.56) | 0.004 | 0.051 |
| Respiratory disease | 1.00 (ref) | 0.79 (0.56-1.13) | 0.75 (0.46-1.20) | 0.23 |  | 1.00 (ref) | 0.90 (0.75-1.08) | 0.86 (0.68-1.08) | 0.31 | 0.27 |
| Pneumonia | 1.00 (ref) | 0.84 (0.57-1.24) | 0.61 (0.36-1.05) | 0.065 |  | 1.00 (ref) | 0.81 (0.66-1.001) | 0.83 (0.64-1.07) | 0.30 | 0.36 |

Supplementary Table 2 (continued)

|  | Q1 (low) | Q3 | Q5 (high) | Trend *P*† |  | Q1 (low) | Q3 | Q5 (high) | Trend *P*^b^ | Interaction *P* |
| --- | --- | --- | --- | --- | --- | --- | --- | --- | --- | --- |
|  | Higher latitude areas^c^ (n = 33,314) | | | |  | Lower latitude areas^d^ (n = 60,371) | | | |  |
| No. of participants | 3923 | 7119 | 8420 |  |  | 14814 | 11618 | 10317 |  |  |
| All-cause mortality | 1.00 (ref) | 0.87 (0.80-0.94) | 0.88 (0.79-0.97) | 0.085 |  | 1.00 (ref) | 0.97 (0.92-1.03) | 0.99 (0.92-1.07) | 0.99 | 0.051 |
| Cancer | 1.00 (ref) | 0.93 (0.82-1.06) | 0.95 (0.80-1.12) | 0.65 |  | 1.00 (ref) | 1.03 (0.93-1.13) | 1.06 (0.93-1.21) | 0.54 | 0.23 |
| Cardiovascular disease | 1.00 (ref) | 0.87 (0.74-1.01) | 0.81 (0.67-0.98) | 0.093 |  | 1.00 (ref) | 0.90 (0.81-1.01) | 0.97 (0.84-1.12) | 0.99 | 0.036 |
| Heart disease | 1.00 (ref) | 0.92 (0.74-1.14) | 0.75 (0.57-0.98) | 0.041 |  | 1.00 (ref) | 0.90 (0.77-1.05) | 0.95 (0.78-1.16) | 0.93 | 0.016 |
| Stroke | 1.00 (ref) | 0.85 (0.67-1.07) | 0.86 (0.64-1.15) | 0.53 |  | 1.00 (ref) | 0.85 (0.70-1.03) | 0.93 (0.73-1.18) | 0.76 | 0.70 |
| Ischemic stroke | 1.00 (ref) | 0.51 (0.32-0.83) | 0.41 (0.23-0.74) | 0.009 |  | 1.00 (ref) | 0.80 (0.57-1.13) | 0.77 (0.50-1.18) | 0.40 | 0.11 |
| Intraparenchymal hemorrhage | 1.00 (ref) | 1.25 (0.82-1.92) | 1.35 (0.79-2.31) | 0.26 |  | 1.00 (ref) | 1.11 (0.77-1.60) | 1.44 (0.89-2.33) | 0.089 | 0.64 |
| Respiratory disease | 1.00 (ref) | 0.84 (0.61-1.14) | 0.89 (0.61-1.30) | 0.88 |  | 1.00 (ref) | 0.88 (0.73-1.07) | 0.80 (0.62-1.02) | 0.076 | 0.82 |
| Pneumonia | 1.00 (ref) | 0.79 (0.56-1.10) | 0.74 (0.49-1.12) | 0.36 |  | 1.00 (ref) | 0.83 (0.67-1.03) | 0.80 (0.60-1.05) | 0.16 | 0.68 |
|  |  |  |  |  |  |  |  |  |  |  |
|  | No history of hypertension (n = 76,184) | | | |  | History of hypertension (n = 17,501) | | | |  |
| No. of participants | 15277 | 15380 | 14798 |  |  | 3460 | 3357 | 3939 |  |  |
| All-cause mortality | 1.00 (ref) | 0.98 (0.93-1.03) | 0.99 (0.92-1.07) | 0.88 |  | 1.00 (ref) | 0.86 (0.78-0.94) | 0.86 (0.77-0.97) | 0.058 | 0.58 |
| Cancer | 1.00 (ref) | 1.04 (0.95-1.14) | 1.03 (0.91-1.15) | 0.99 |  | 1.00 (ref) | 0.89 (0.75-1.05) | 1.04 (0.84-1.29) | 0.60 | 0.15 |
| Cardiovascular disease | 1.00 (ref) | 0.97 (0.87-1.08) | 0.99 (0.86-1.14) | 0.76 |  | 1.00 (ref) | 0.79 (0.68-0.93) | 0.78 (0.64-0.94) | 0.017 | 0.49 |
| Heart disease | 1.00 (ref) | 0.99 (0.85-1.16) | 1.00 (0.82-1.22) | 0.64 |  | 1.00 (ref) | 0.79 (0.64-0.98) | 0.67 (0.51-0.87) | 0.002 | 0.26 |
| Stroke | 1.00 (ref) | 0.90 (0.75-1.08) | 0.96 (0.76-1.21) | 0.99 |  | 1.00 (ref) | 0.78 (0.61-1.01) | 0.81 (0.59-1.10) | 0.30 | 0.90 |
| Ischemic stroke | 1.00 (ref) | 0.70 (0.49-1.01) | 0.66 (0.43-1.03) | 0.12 |  | 1.00 (ref) | 0.70 (0.45-1.09) | 0.58 (0.33-1.02) | 0.13 | 0.36 |
| Intraparenchymal hemorrhage | 1.00 (ref) | 1.14 (0.82-1.59) | 1.33 (0.87-2.05) | 0.13 |  | 1.00 (ref) | 1.30 (0.79-2.16) | 1.49 (0.79-2.80) | 0.23 | 0.45 |
| Respiratory disease | 1.00 (ref) | 0.90 (0.74-1.09) | 0.94 (0.73-1.20) | 0.77 |  | 1.00 (ref) | 0.83 (0.62-1.11) | 0.66 (0.45-0.96) | 0.040 | 0.087 |
| Pneumonia | 1.00 (ref) | 0.88 (0.70-1.09) | 0.92 (0.70-1.21) | 0.81 |  | 1.00 (ref) | 0.71 (0.51-0.98) | 0.56 (0.37-0.84) | 0.007 | 0.079 |

Supplementary Table 2 (continued)

|  | Q1 (low) | Q3 | Q5 (high) | Trend *P*† |  | Q1 (low) | Q3 | Q5 (high) | Trend *P*^b^ | Interaction *P* |
| --- | --- | --- | --- | --- | --- | --- | --- | --- | --- | --- |
|  | Calcium intake < median (n = 46,842) | | | |  | Calcium intake ≥ median (n = 46,843) | | | |  |
| No. of participants | 12660 | 8854 | 7334 |  |  | 6077 | 9883 | 11403 |  |  |
| All-cause mortality | 1.00 (ref) | 0.96 (0.91-1.03) | 0.97 (0.89-1.06) | 0.57 |  | 1.00 (ref) | 0.91 (0.85-0.98) | 0.91 (0.84-0.997) | 0.19 | 0.12 |
| Cancer | 1.00 (ref) | 1.01 (0.91-1.12) | 0.98 (0.85-1.14) | 0.49 |  | 1.00 (ref) | 0.97 (0.86-1.10) | 1.04 (0.90-1.21) | 0.39 | 0.97 |
| Cardiovascular disease | 1.00 (ref) | 0.92 (0.82-1.05) | 0.99 (0.84-1.16) | 0.87 |  | 1.00 (ref) | 0.88 (0.77-1.01) | 0.82 (0.70-0.97) | 0.045 | 0.31 |
| Heart disease | 1.00 (ref) | 0.93 (0.79-1.11) | 1.03 (0.82-1.29) | 0.53 |  | 1.00 (ref) | 0.91 (0.76-1.10) | 0.75 (0.60-0.94) | 0.007 | 0.12 |
| Stroke | 1.00 (ref) | 0.88 (0.72-1.07) | 0.88 (0.68-1.14) | 0.41 |  | 1.00 (ref) | 0.83 (0.66-1.04) | 0.87 (0.67-1.14) | 0.74 | 0.94 |
| Ischemic stroke | 1.00 (ref) | 0.75 (0.52-1.09) | 0.44 (0.26-0.74) | 0.008 |  | 1.00 (ref) | 0.61 (0.40-0.95) | 0.73 (0.45-1.19) | 0.55 | 0.69 |
| Intraparenchymal hemorrhage | 1.00 (ref) | 1.06 (0.74-1.54) | 1.55 (0.97-2.49) | 0.065 |  | 1.00 (ref) | 1.25 (0.81-1.94) | 1.15 (0.67-1.97) | 0.50 | 0.63 |
| Respiratory disease | 1.00 (ref) | 0.87 (0.70-1.08) | 0.89 (0.67-1.19) | 0.48 |  | 1.00 (ref) | 0.86 (0.67-1.10) | 0.74 (0.55-1.004) | 0.11 | 0.13 |
| Pneumonia | 1.00 (ref) | 0.81 (0.64-1.04) | 0.84 (0.60-1.16) | 0.28 |  | 1.00 (ref) | 0.82 (0.62-1.08) | 0.71 (0.51-0.996) | 0.12 | 0.36 |

Abbreviation: ref, reference.

^a^Adjusted for age (year), sex, study area (11 areas), body mass index (<21, 21–22.9, 23–24.9, 25–26.9, or ≥27 kg/m^2^), smoking status (never, past, or current with a consumption of <20 or ≥20 cigarettes/day), alcohol consumption (nondrinker, occasional drinker, or drinker with a consumption of <150, 150–299, 300–449, or ≥450 g ethanol/week), history of diabetes mellitus (yes or no), history of hypertension (yes or no), total physical activity (quartile of metabolic equivalent task hours/day), occupation (agriculture, forestry, or fishery; salaried, self-employed, or professional; or housework, unemployed, or retired), leisure-time physical activity (<1 time/month, 1–2 times/month, or ≥1 time/week), total energy intake (kcal/day), supplement use (yes or no), green tea consumption (almost never, <1, 1, 2–3, or ≥4 cups/day), coffee consumption (almost never, <1, 1, or ≥2 cups/day), energy-adjusted calcium intake (mg/day), and n-3 polyunsaturated fatty acid intake (%energy). In stratified analysis by sex, sex was excluded from adjustment factors. In stratified analysis by history of hypertension, history of hypertension was excluded from adjustment factors.

^b^Based on Cox proportional hazards model, assigning median intake to the quintile of vitamin D intake.

^c^Iwate, Akita, Nagano, and Niigata.

^d^Okinawa Chubu, Tokyo, Ibaraki, Kochi, Nagasaki, Okinawa Miyako, Osaka.
